# microRNAs: a role in drug resistance in parasitic nematodes?

**DOI:** 10.1016/j.pt.2010.05.003

**Published:** 2010-09

**Authors:** Eileen Devaney, Alan D. Winter, Collette Britton

**Affiliations:** Parasitology Group, Division of Veterinary Infection and Immunity, Institute for Comparative Medicine, School of Veterinary Medicine, University of Glasgow, Bearsden Road, Glasgow G61 1QH, UK

## Abstract

Drug resistance in parasitic nematodes is an increasing problem worldwide, with resistance reported to all three commonly used classes of anthelmintics. Most studies to date have sought to correlate the resistant phenotype with genotypic changes in putative target molecules. Although this approach has identified mutations in several relevant genes, resistance might result from a complex interaction of different factors. Here we propose an alternative mechanism underlying the development of drug resistance based on functional differences in microRNA activity in resistant parasites. microRNAs play an important role in resistance to chemotherapeutic agents in many tumour cells and here we discuss whether they might also be involved in anthelmintic resistance in parasitic nematodes.

## The problem of drug resistance in parasitic nematodes

Parasitic nematodes of humans and animals remain a major problem in many areas of the world. Control of these parasites relies almost exclusively on the use of chemotherapeutic agents, of which three classes are in common use. These are the tetrahydropyrimidines/imidazothiazoles (e.g. pyrantel, levamisole), the benzimidazoles (e.g. fenbendazole) and the macrocyclic lactones (e.g. ivermectin, moxidectin). Whereas most drugs retain efficacy against human parasitic nematodes, there are indications that resistance to ivermectin might be developing in the filarial nematode *Onchocerca volvulus* [1]. By contrast, the problem of drug resistance in veterinary nematodes is widespread, particularly in parasites of sheep, goats and horses [2] and, more recently, cattle [3]. For sheep parasites such as *Teladorsagia circumcincta* and *Haemonchus contortus*, resistance has been reported to all three classes of drug and, indeed, triple-resistant parasites exist in parts of the world, including Scotland [4]. As well as causing considerable economic loss (an estimated £84 million per annum to the sheep industry in the UK [5]), these pathogens cause significant welfare problems in infected animals. In the short term, the availability of novel anthelmintics, such as the amino-acetonitrile derivative (AAD) [6], monepantel, marketed as Zolvix, offer temporary relief. Yet, by analogy with how quickly resistance has developed to the current compounds, it is probably simply a matter of time before the efficacy of novel compounds is compromised.

Understanding the mode of action of anthelmintics and the mechanisms of resistance is important in terms of attempting to monitor and modulate the resistant phenotype. Much of our knowledge of mechanisms of drug action and the likely genes involved in resistance comes from studies on *Caenorhabditis elegans* [7], where the relative ease of generating resistant mutants and identifying mutations has facilitated such studies. This has been most clearly demonstrated for the β-tubulin gene *ben-1*, in which mutations conferred resistance to benzimidazole (BZ) compounds in *C. elegans* [8]. In *H. contortus*, mutations in the β-tubulin isotype 1 gene (*Hc-iso-1*) are also associated with BZ resistance. Transformation of BZ-resistant *C. elegans* with the wild-type *Hc-iso-1* allele restores sensitivity to BZ compounds, whereas transformation with the mutant *Hc-iso-1* gene had no such effect [9]. These results helped establish that β-tubulin isotype-1 mutations are a major determinant of BZ resistance in parasitic nematodes. Confirming a role for specific gene mutations in resistance to other anthelmintics has, however, proven more difficult. For example, studies in *C. elegans* have indicated that resistance to ivermectin might be polygenic and that mutations at any one contributing locus may be insufficient for a fully resistant phenotype [10]. It has been suggested that a similar scenario might exist in parasitic nematodes [11]. The situation is confounded by the significant genetic polymorphism of parasite populations, particularly for trichostrongylid species [12], making it difficult to conclude that resistance is due to a particular polymorphism, or whether other linked mutations might be responsible.

Recent studies on possible mechanisms of drug resistance in parasitic nematodes have identified mutations within the coding regions or the promoters of candidate genes first identified in *C. elegans* ([11,13–15]). Nevertheless, by analogy with other eukaryotes, drug resistance in nematodes could result from a range of different mechanisms in addition to functional mutations in specific genes. These include altered levels of expression of target or non-target genes, such as drug transporters or detoxification enzymes. Changes in the expression of such genes could arise via a number of different processes such as epigenetic modifications in chromatin or by a variety of transcriptional or post-transcriptional mechanisms, including trans-splicing and capping [16]. In tumour cells, one specific type of post-transcriptional regulation, the alteration of microRNA (miRNA) expression or activity, is increasingly implicated in drug resistance. In this article we discuss whether miRNAs are involved in drug resistance in parasitic nematodes.

## miRNAs

miRNAs are one of a family of endogenous small non-coding RNAs, including small interfering RNAs (siRNAs) and piwi-interacting RNAs (piRNAs), that have been identified in a diverse range of organisms [17]. They were first identified in *C. elegans* as key components of the heterochronic pathway, the mechanism by which developmental timing is regulated in the worm [18,19]. miRNAs are the focus of intense interest given their important roles in the control of gene expression in many biological and pathological processes, such as cell and organ development, differentiation and homeostasis, tumour suppression and stem cell regulation [20]. The biogenesis of miRNAs (Box 1) and the mechanisms by which they exert their regulatory function are increasingly understood [21]. miRNAs regulate the expression of target genes at the post-transcriptional level by base pairing with defined sites often, but not exclusively, located in the 3′-UTR of target genes. In animal cells, specific miRNAs have been shown to repress translation, whereas others appear to act by inducing degradation of the target mRNA. Since their discovery in *C. elegans*, miRNAs have been identified in a diverse range of plants and animals and appear to be abundant in most genomes; the current version (release 15) of miRBase (http://www.mirbase.org/) identifies 940 miRNAs in humans and 175 in *C. elegans*. In addition, up to one third of all human mRNAs might be regulated by miRNAs [22]. Now that genome sequence data are available for several species of parasitic nematode [23], it is possible to identify miRNAs in parasitic species and to investigate their roles in the biology of parasites. For example, a number of important miRNAs including *let-7* and *lin-4* have been identified in the *B. malayi* genome [24].

## miRNAs and drug resistance in tumour cells

**Figure 1:**
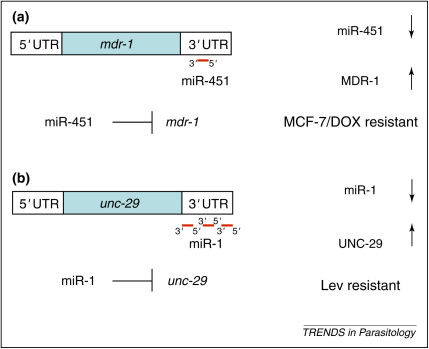
Potential mechanisms for miRNA involvement in drug resistance. **(a)** In human MCF-7 breast cancer cells, miR-451 negatively regulates translation of the P-glycoprotein gene *mdr-1*, via a complementary miR-451 site in the 3′-UTR of *mdr-1.* In MCF-7 cells resistant to doxorubicin (MCF-7/DOX), decreased levels of miR-451 result in an increased level of MDR-1 protein with resultant DOX resistance [28]. **(b)** In *C. elegans,* miR-1 negatively regulates the *unc-29* nAChR subunit via three complementary sites in the *unc-29* 3′UTR. In *mir-1* null mutants, UNC-29 protein level is increased, which alters nAChR composition, leading to a decreased sensitivity to levamisole [37].

miRNAs have been the focus of much interest in tumour cell biology because they appear to play a role in the initiation and progress of some cancers and indeed are considered by some to function as oncogenes or tumour suppressors [25]. In addition, many tumour cells express an altered profile of miRNAs, which might have diagnostic and/or prognostic potential [26]. Furthermore, expression profiling of drug-sensitive and drug-resistant tumour cell lines has suggested a role for miRNAs in the development of drug resistance [27]. For example, in the breast cancer cell line MCF-7, doxyrubicin resistance appears to be associated with significant changes in the levels of specific miRNAs [28]. These cells express very high levels of P-glycoprotein (encoded by the *mdr-1* gene), an important mechanism of drug efflux. The 3′-UTR of the *mdr-1* gene contains a binding site for miR-451, which negatively regulates *mdr-1* expression (Figure 1(a)). It was shown that transfection of doxyrubicin-resistant cells with this miRNA resulted in decreased levels of P-glycoprotein and a 2.5-fold increase in sensitivity to doxyrubicin.

A number of recent studies have further demonstrated that drug metabolising enzymes and transporters can be post-transcriptionally regulated by miRNAs and, importantly, that changes in expression of many miRNAs are induced by drug treatment [29]. For example, miR-328 is downregulated ten-fold in xenograft tumours following gemcitabine treatment [30]. miR-328 is involved in the negative regulation of the ABCG2 efflux transporter [31] and therefore downregulation of miR-328 results in overexpression of the transporter and multidrug resistance [30].

As well as altered expression of miRNAs leading to drug resistance, deletions or mutations within the binding targets of miRNAs can affect miRNA function. A single nucleotide polymorphism (SNP) near the binding site of miR-24 in the 3′-UTR of the human dihyrofolate reductase (DHFR) gene prevents repression by miR-24, resulting in overexpression of DHFR and resistance to methotrexate [32]. Indeed mutations within 3′-UTR binding sites occur at a higher level than SNPs in miRNA sequences themselves [33], probably because miRNAs have multiple targets, and therefore miRNA mutation is likely to lead to more serious consequences. In a different system, several drug resistant cell lines that overexpress ABCG2 were shown to contain truncated 3′-UTRs. The deleted region contains a binding site for miR-519c, and the absence of this binding site in resistant cells leads to overexpression of ABCG2 and drug resistance [34]. The accumulating data on miRNA and mRNA expression and drug resistance in cancer cells are now being integrated to predict responses to drugs and improve treatment efficiency, an area referred to as ‘miRNA pharmacogenomics’ [35].

It is possible that a link between miRNAs and drug resistance is more likely to occur in cancer cells, as the cancerous state can itself be due to miRNA mis-expression. However, the study of miRNAs in cancer has clearly established that miRNA-mediated alterations in levels of drug targets, drug transporters, metabolic enzymes or cell apoptosis proteins can lead to drug resistance. Altered gene expression associated with drug resistance in other systems indicates that examination of miRNA activity and 3′-UTR interactions in parasitic nematodes is warranted to improve our understanding of drug resistance mechanisms.

## Could miRNAs have a role in drug resistance in nematodes?

The study of miRNAs and their functions in parasitic nematodes is in its infancy, although much is known about specific miRNAs in *C. elegans* [36]. Recent work on this nematode indicates that miRNAs might play a role in drug resistance, and indicates that this area is ripe for investigation in parasitic nematodes. In *C. elegans*, miR-1 negatively regulates the expression of two nicotinic acetylcholine receptor (nAChR) subunits, *unc-29* and *unc-63* [37]. Intriguingly, in *mir-1* mutants the expression of both UNC-29 and UNC-63 subunits is increased, and this corresponds with decreased muscle sensitivity to acetylcholine and levamisole (Figure 1(b)). It is perhaps surprising that an increased level of particular AChR subunits leads to an altered response to levamisole. Nevertheless, it was speculated that altering the subunit composition of the AChR could affect receptor biogenesis or function and that miRNAs can alter the composition and activity of other heteromultimeric receptors [37]. These findings are consistent with changes in kinetics and affinity of mammalian nAChRs that follow changes in subunit composition [38] and are supported by studies on the *Ascaris suum* nAChR which highlighted the importance of subunit composition for drug sensitivity [39].

Interestingly, in a very different approach using immunoprecipitation to isolate miRNA-target complexes in *C. elegans* during development, mRNAs encoding ion channels and receptors were particularly enriched in the data set [36]. Since these types of proteins are well represented among known drug targets in nematodes, miRNAs might well be involved in altering their expression and activity. Recently, two studies in parasitic nematode species reported alterations in expression levels of receptors and ion channels in drug resistant isolates. Following pyrantel treatment, changes in transcript levels of the nAChR genes *unc-29, unc-38* and *unc-63* were found in isolates of the dog hookworm *Ancylostoma caninum* [40]. In this case a highly resistant isolate showed diminished expression of the three receptor subunit genes compared to an isolate with a low level of pyrantel resistance. Whether the lower transcript level of any of these three genes corresponds with an increase in regulatory miRNA expression or activity has not been examined, but would be of great interest. Similarly, isolates of *H. contortus* resistant to ivermectin were found to have lowered expression of a novel ligand-gated ion channel gene (*Hc-GGR3*) [41]. Interestingly, a SNP was identified in the *Hc-GGR3* 3′-UTR of resistant worms. It was proposed that this polymorphism might be associated with resistance, although functional analysis is required to test this. Given the high degree of conservation of some miRNA sequences across species, including the miR-1 sequence, *C. elegans* presents a suitable model system in which to functionally test potential miRNA-3′-UTR interactions, which cannot readily be examined directly in parasitic species.

As well as changes in expression levels of potential drug targets, alterations in the expression of drug transporters have been reported in parasitic nematodes following anthelmintic treatment. This might be analogous to the changes in transporter levels in tumour cells exposed to drugs, as discussed previously. In *H. contortus* and *O. volvulus*, treatment with ivermectin results in a reduction in allele frequency and overexpression of P-glycoprotein and other ABC transporter genes [42]. Similarly, two independent isolates of *H. contortus* that were selected for BZ resistance show an elevated frequency of the same P-glycoprotein allele [14]. Although different drug classes can interact with different ABC transporters, these findings indicate that the mechanism underlying this allele selection might be the same and have implications for the development of resistance to new drug classes. A recent study in *C. elegans* also found that exposure to increasing concentrations of ivermectin resulted in increased expression of several P-glycoprotein and ABC transporter genes [43]. It would obviously be interesting to compare the 3′-UTR region of P-glycoprotein genes from susceptible and resistant nematodes for regulation by miRNAs.

## Future perspectives

Herein the possibility that changes in gene expression levels associated with drug resistance in parasitic nematodes are mediated by miRNAs has been discussed. This might be a mechanism of altering drug efficacy; given the link between miRNAs and drug responses in cancer cells, this area is ripe for detailed investigation. With the number of genome sequencing projects underway, miRNAs present in parasitic nematodes can be identified using both *in silico* approaches [44] (for example by combining BLAST [45] and RNAfold [46] analysis to search for homologues of known miRNAs), and small RNA sequencing [24]. Potential target mRNAs can be identified using available bioinformatics programmes such as MiRanda [47] and PicTar [48], combined with immunoprecipitation of mRNAs in RISC complexes and sequencing [49]. A number of potential target mRNAs relevant to drug resistance in parasitic nematodes have been discussed above; bioinformatic and biochemical approaches can be used to determine whether these are likely to be real targets of miRNA regulation. A potential interaction between a miRNA and the 3′-UTR of a target gene from a parasitic nematode could then be verified using *C. elegans* as a heterologous expression system*.*

Currently, the potential correlation between antihelmintic resistance and changes in particular gene sequence or expression level has been limited to the study of a small number of candidate genes. Examining and comparing miRNA levels, as well as mRNA expression in drug resistant and susceptible isolates, through the use of microarrays will produce a global picture of changes that might correlate with drug resistance. In addition, miRNA inhibitors and mimics (Box 2) are available and can be used to examine the upregulation or downregulation of miRNAs and their potential mRNA targets. Could these miRNA inhibitors or mimics be effective in reversing drug resistance in nematodes?

## Figures and Tables

**Figure I:**
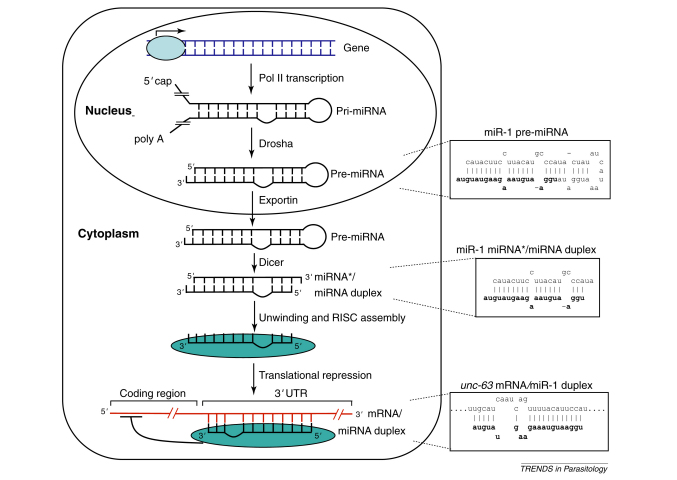


## Box 1 Biogenesis of miRNAs

miRNAs are transcribed as hairpin precursors, which are sequentially processed by the RNase III enzymes Drosha and Dicer to imperfectly paired double-stranded intermediates. One strand of the duplex is then selectively assembled into a cytoplasmic protein complex, known as an RNA-induced silencing complex (RISC). Protein coding mRNAs which contain complementary sequences to the miRNA (usually in their 3′-UTR) are bound by this complex, most often resulting in translational repression in animals. An example of *C. elegans* miR-1 processing is presented on the RHS of the diagram, with the mature miRNA sequence shown in bold (Figure I).

## Box 2 miRNA modulation

Strategies to modulate miRNA levels in cells and organisms have been developed both to investigate miRNA function experimentally and for potential therapeutic application. Although genetic mutant analysis is most reliably informative for functional miRNA studies, generating mutants is time consuming and not possible with parasitic nematodes. miRNA levels, unlike protein-coding mRNAs, cannot be knocked-down using RNAi. However, miRNA function has been investigated by disruption using chemically engineered oligonucleotides as competitive inhibitors. These anneal to the mature RISC-bound miRNA by anti-sense complementarity and compete for binding to the natural mRNA target. Repression is therefore removed and target mRNAs become available for translation. Inhibitory oligonucleotides have been designed bearing various distinct modifications, such as 2′-*O*-methyl oligonucleotides (known as antagomirs) [50] or locked nucleic acid (LNA) oligonucleotides [51]. These modifications result in enhanced binding affinity for target sequences and prevent degradation of the oligonucleotide by RISC or other cellular ribonucleases. Antisense oligonucleotides have been used successfully in cultured cells [52,53], *Drosophila* embryos [54], *C. elegans* [55], mouse [50] and in non-human primates [56]. Multiple miRNAs can be disrupted simultaneously using mixes of inhibitory oligonucleotides. To ensure that effects are due only to miRNA inhibition, experiments should be controlled using antisense oligonucleotides with scrambled or mis-matched sequences.

In cultured cells, delivery of antisense oligonucleotides is usually accomplished by lipid transfection or electroporation, whereas micro-injection is used for *Drosophila* and *C. elegans*. In *C. elegans* this is currently performed by injection of early larval stage worms (a technically difficult process), with the effect determined in the injected animal itself [55]. Techniques to allow inheritance of the inhibitory oligonucleotides have been developed recently [57]. Uptake of a fluorescently-labelled inhibitory oligonucleotide by soaking larvae of the filarial nematode *Brugia pahangi* (Devaney, E., and Britton, C., unpublished data) indicates that the approaches described above for other organisms could be adapted for the study of parasitic nematode miRNAs.

An alternative to introducing antisense oligonucleotide is high level expression of transgenes carrying multiple sequences complementary to the miRNA (‘miRNA sponges’) [58]. Here partial complementarity is used so that sequences are not cleaved; however, this may result in multiple miRNAs being targeted. Although more time-consuming, delivery or uptake are not issues with this approach and it has recently been adapted to provide tissue-specific and temporal control over miRNA disruption in *Drosophila* [59]. Transgenic miRNA inhibition is not currently applicable to parasitic nematodes but could be adapted for use in *C. elegans*. miRNA function can also be investigated by upregulating their expression, accomplished either by transgenically overexpressing the miRNA, as performed in cultured cells [60] and *C. elegans* [61], or by introducing synthetic miRNAs (miRNA mimics). In overexpression studies, an opposing effect on target mRNA or protein level to that produced by inhibition would provide strong evidence of a true target.

## References

[1] Bourguinat C. (2008). P-glycoprotein-like protein, a possible genetic marker for ivermectin resistance selection in Onchocerca volvulus. Mol. Biochem. Parasitol..

[2] Kaplan R.M. (2004). Drug resistance in nematodes of veterinary importance: a status report. Trends Parasitol..

[3] Gasbarre L.C. (2009). The identification of cattle nematode parasites resistant to multiple classes of anthelmintics in a commercial cattle population in the US. Vet. Parasitol..

[4] Sargison N. (2001). Multiple anthelmintic resistance in sheep. Vet. Record.

[5] Nieuwhof G.J., Bishop S.C. (2005). Costs of the major endemic diseases of sheep in Great Britain and the potential benefits of reduction in disease impact. Anim. Sci..

[6] Kaminsky R. (2008). A new class of anthelmintics effective against drug-resistant nematodes. Nature.

[7] Gilleard J.S. (2006). Understanding anthelmintic resistance: the need for genomics and genetics. Int. J. Parasitol..

[8] Driscoll M. (1989). Genetic and molecular analysis of a *Caenorhabditis elegans* beta-tubulin that conveys benzimidazole sensitivity. J. Cell Biol..

[9] Kwa M.S. (1995). Beta-tubulin genes from the parasitic nematode Haemonchus contortus modulate drug resistance in *Caenorhabditis elegans*. J. Mol. Biol..

[10] Dent J.A. (2000). The genetics of ivermectin resistance in *Caenorhabditis elegans*. Proc. Natl. Acad. Sci. USA.

[11] McCavera S. (2007). Nematode ligand-gated chloride channels: an appraisal of their involvement in macrocyclic lactone resistance and prospects for developing molecular markers. Parasitology.

[12] Redman E. (2008). Microsatellite analysis reveals marked genetic differentiation between *Haemonchus contortus* laboratory isolates and provides a rapid system of genetic fingerprinting. Int. J. Parasitol..

[13] Von Samson-Himmelstjerna G. (2007). Single nucleotide polymorphism (SNP) markers for benzimidazole resistance in veterinary nematodes. Parasitology.

[14] Blackhall W.J. (2008). P-glycoprotein selection in strains of *Haemonchus**contortus* resistant to benzimidazoles. Vet. Parasitol..

[15] Rufener, L. *et al.* (2009) *Haemonchus**contortus* acetylcholine receptors of the DEG-3 subfamily and their role in sensitivity to monepantel. *PLoS Pathog.* 5, e1000380 (www.plospathogens.org). 10.1371/journal.ppat.1000380 PMC2662886 19360096

[16] Cheng G. (2007). *In vivo* translation and stability of trans-spliced mRNAs in nematode embryos. Mol. Biochem. Parasitol..

[17] Ghildiyal M., Zamore P.D. (2009). Small silencing RNAs: an expanding universe. Nat. Rev. Genet..

[18] Lee R.C. (1993). The *C.**elegans* heterochronic gene lin-4 encodes small RNAs with antisense complementarity to lin-14. Cell.

[19] Reinhart B.J. (2000). The 21-nucleotide let-7 RNA regulates developmental timing in *Caenorhabditis**elegans*. Nature.

[20] Pasquinelli A.E. (2005). MicroRNAs: a developing story. Curr. Opin. Genet. Dev..

[21] Chekulaeva M., Filipowicz W. (2009). Mechanisms of miRNA-mediated post-transcriptional regulation in animal cells. Curr. Opin. Cell Biol..

[22] Lewis B.P. (2003). Prediction of mammalian microRNA targets. Cell.

[23] Ghedin E. (2007). Draft genome of the filarial nematode parasite *Brugia**malayi*. Science.

[24] Poole C.B. (2009). Cloning and bioinformatic identification of small RNAs in the filarial nematode. Brugia malayi. Mol. Biochem. Parasitol..

[25] Zhang B. (2007). microRNAs as oncogenes and tumor suppressors. Dev. Biol..

[26] Lu J. (2005). MicroRNA expression profiles classify human cancers. Nature.

[27] Zheng T. (2010). Role of microRNA in anticancer drug resistance. Int. J. Cancer.

[28] Kovalchuk O. (2008). Involvement of microRNA-451 in resistance of the MCF-7 breast cancer cells to chemotherapeutic drug doxorubicin. Mol. Cancer Ther..

[29] Yu A.M. (2009). Role of microRNAs in the regulation of drug metabolism and disposition. Expert Opin. Drug Metab. Toxicol..

[30] Meng F. (2006). Involvement of human micro-RNA in growth and response to chemotherapy in human cholangiocarcinoma cell lines. Gastroenterology.

[31] Pan Y.Z. (2009). MicroRNA-328 negatively regulates the expression of breast cancer resistance protein (BCRP/ABCG2) in human cancer cells. Mol. Pharmacol..

[32] Mishra P.J. (2007). A miR-24 microRNA binding-site polymorphism in dihydrofolate reductase gene leads to methotrexate resistance. Proc. Natl. Acad. Sci. U. S. A..

[33] Saunders M.A. (2007). Human polymorphism at microRNAs and microRNA target sites. Proc. Natl. Acad. Sci. USA.

[34] To K.K. (2009). Escape from hsa-miR-519c enables drug-resistant cells to maintain high expression of ABCG2. Mol. Cancer Ther..

[35] Bertino J.R. (2007). Pharmacogenomics of microRNA: a miRSNP towards individualized therapy. Pharmacogenomics.

[36] Zhang L. (2009). Systematic analysis of dynamic miRNA-target interactions during C. elegans development. Development.

[37] Simon D.J. (2008). The microRNA miR-1 regulates a MEF-2-dependent retrograde signal at neuromuscular junctions. Cell.

[38] Millar N.S. (2003). Assembly and subunit diversity of nicotinic acetylcholine receptors. Biochem. Soc. Trans..

[39] Williamson, S.M. *et al.* (2009) The nicotinic acetylcholine receptors of the parasitic nematode *Ascaris**suum*: formation of two distinct drug targets by varying the relative expression levels of two subunits. *PLoS Pathog.* 5, e1000517 (www.plospathogens.org). 10.1371/journal.ppat.1000517 PMC2705655 19609360

[40] Kopp S.R. (2009). Acetylcholine receptor subunit genes from *Ancylostoma**caninum*: altered transcription patterns associated with pyrantel resistance. Int. J. Parasitol..

[41] Rao V.T. (2009). A dopamine-gated ion channel (HcGGR3*) from *Haemonchus**contortus* is expressed in the cervical papillae and is associated with macrocyclic lactone resistance. Mol. Biochem. Parasitol..

[42] Prichard R.K., Roulet A. (2007). ABC transporters and beta-tubulin in macrocyclic lactone resistance: prospects for marker development. Parasitology.

[43] James C.E., Davey M.W. (2009). Increased expression of ABC transport proteins is associated with ivermectin resistance in the model nematode *Caenorhabditis**elegans*. Int. J. Parasitol..

[44] Mendes N.D. (2009). Current tools for the identification of miRNA genes and their targets. Nucleic Acids Res..

[45] Altschul S.F. (1990). Basic local alignment search tool. J. Mol. Biol..

[46] Hofacker I.L. (1994). Fast folding and comparison of RNA secondary structures. Monatshefte Fur Chemie.

[47] John, B. *et al.* (2004) Human MicroRNA targets. *PLoS Biol.* 2, e363 (www.plosbiology.org). 10.1371/journal.pbio.0020363 PMC521178 15502875

[48] Lall S. (2006). A genome-wide map of conserved microRNA targets in *C. elegans*. Curr. Biol..

[49] Hsu, R.J. *et al.* (2009) Labeled microRNA pull-down assay system: an experimental approach for high-throughput identification of microRNA-target mRNAs. *Nucleic Acids Res.* 37, e77 (http://nar.oxfordjournals.org/). 10.1093/nar/gkp274 PMC2691847 19420057

[50] Krutzfeldt J. (2005). Silencing of microRNAs in vivo with ‘antagomirs’. Nature.

[51] Orom U.A. (2006). LNA-modified oligonucleotides mediate specific inhibition of microRNA function. Gene.

[52] Esau C.C. (2008). Inhibition of microRNA with antisense oligonucleotides. Methods.

[53] Horwich M.D., Zamore P.D. (2008). Design and delivery of antisense oligonucleotides to block microRNA function in cultured Drosophila and human cells. Nat. Protoc..

[54] Leaman D. (2005). Antisense-mediated depletion reveals essential and specific functions of microRNAs in Drosophila development. Cell.

[55] Hutvagner, G. *et al.* (2004) Sequence-specific inhibition of small RNA function. *PLoS Biol.* 2, E98 (www.plosbiology.org). 10.1371/journal.pbio.0020098 PMC350664 15024405

[56] Elmen J. (2008). LNA-mediated microRNA silencing in non-human primates. Nature.

[57] Zheng, G. *et al.* (2010) Inhibiting miRNA in *Caenorhabditis**elegans* using a potent and selective antisense reagent. *Silence* 1, 9. 10.1186/1758-907X-1-9 PMC2864223 20359322

[58] Ebert M.S. (2007). MicroRNA sponges: competitive inhibitors of small RNAs in mammalian cells. Nat. Methods.

[59] Loya C.M. (2009). Transgenic microRNA inhibition with spatiotemporal specificity in intact organisms. Nat. Methods.

[60] Zeng Y. (2002). Both natural and designed micro RNAs can inhibit the expression of cognate mRNAs when expressed in human cells. Mol. Cell.

[61] Johnson S.M. (2005). RAS is regulated by the let-7 microRNA family. Cell.

